# Supplementary material for: The effect of prone positioning on maternal haemodynamics and fetal wellbeing in the third trimester–A primary cohort study with a scoping review
Source: PLoS One. 2023 Oct 11;18(10):e0287804. doi: 10.1371/journal.pone.0287804 (PMC10566740; doi:10.1371/journal.pone.0287804)
Supplement: S1 Checklist — (DOCX) [file pone.0287804.s001.docx]

**MOOSE Checklist**

From: [Donna F. Stroup](http://jama.ama-assn.org/search?author1=Donna+F.+Stroup&sortspec=date&submit=Submit), PhD, MSc; [Jesse A. Berlin](http://jama.ama-assn.org/search?author1=Jesse+A.+Berlin&sortspec=date&submit=Submit), ScD; [Sally C. Morton](http://jama.ama-assn.org/search?author1=Sally+C.+Morton&sortspec=date&submit=Submit), PhD; [Ingram Olkin](http://jama.ama-assn.org/search?author1=Ingram+Olkin&sortspec=date&submit=Submit), PhD; [G. David Williamson](http://jama.ama-assn.org/search?author1=G.+David+Williamson&sortspec=date&submit=Submit), PhD; [Drummond Rennie](http://jama.ama-assn.org/search?author1=Drummond+Rennie&sortspec=date&submit=Submit), MD; [David Moher](http://jama.ama-assn.org/search?author1=David+Moher&sortspec=date&submit=Submit), MSc; [Betsy J. Becker](http://jama.ama-assn.org/search?author1=Betsy+J.+Becker&sortspec=date&submit=Submit), PhD; [Theresa Ann Sipe](http://jama.ama-assn.org/search?author1=Theresa+Ann+Sipe&sortspec=date&submit=Submit), PhD; [Stephen B. Thacker](http://jama.ama-assn.org/search?author1=Stephen+B.+Thacker&sortspec=date&submit=Submit), MD, MSc; for the Meta-analysis Of Observational Studies in Epidemiology (MOOSE) Group. **Meta-analysis of Observational Studies in Epidemiology. A Proposal for Reporting** JAMA. 2000;283(15):2008-2012. doi: 10.1001/jama.283.15.2008

|  | Reported on page | Comments |
| --- | --- | --- |
| **Reporting of background should include** | | |
| Problem definition | 5 | Within introduction section of manuscript |
| Hypothesis statement | 5 | Within last paragraph of the introduction. |
| Description of study outcomes | 5 | Within last paragraph of the introduction. |
| Type of exposure or intervention used | 5 | Within last paragraph of the introduction. |
| Type of study designs used | 5 | Scoping review was part of a larger piece of work. |
| Study population | 5 | Clinical question described in last paragraph of the introduction section. |
| **Reporting of search strategy should include** | | |
| Qualifications of searchers (e.g. librarians and investigators) | 10 | Initials of relevant researchers at the beginning of methods section. |
| Search strategy, including time period used in the synthesis and key words | 10 and Suppl 1 | Example search strategy included. |
| Effort to include all available studies, including contact with authors | 10 | Included grey literature and libraries of theses. |
| Databases and registries searched | 10 | Cochrane (Database of Systematic Reviews; Central Register of Controlled Trials), Medline and Embase. |
| Search software used, name and version, including special features used (e.g. explosion) | Suppl 1 | Example search strategy included. No specific software used. |
| Use of hand searching (eg reference lists of obtained articles) | 10 | Statement regarding the use of hand-searching in methods section. |
| List of citations located and those excluded, including justification | Suppl 2 and Suppl 3 | Citations included. As this was a scoping review with meta-analysis no studies were excluded. |
| Method of addressing articles published in languages other than English | N/A | We did not identify any non-English articles in our search strategy. |
| Method of handling abstracts and unpublished studies | 10 | Included grey literature and libraries of theses. No studies were excluded. |
| Description of any contact with authors | N/A | No authors were contacted for additional information. |
| **Reporting of methods should include** | | |
| Description of relevance or appropriateness of studies assembled for assessing the hypothesis to be tested | 11 |  |
| Rationale for the selection and coding of data (e.g. sound clinical principles or convenience) | 11 |  |
| Documentation of how data were classified and coded (eg multiple raters, blinding and interrater reliability) | 11 | Data were extracted from primary publications. No classification was required. |
| Assessment of confounding (eg comparability of cases and controls in studies where appropriate) | N/A | Case-control studies would not be a suitable means to assess the haemodynamic changes of maternal prone position. |
| Assessment of study quality, including blinding of quality assessors, stratification or regression on possible predictors of study results | N/A | As this was a scoping review quality assessment was not recommended. |
| Assessment of heterogeneity | 11 | Used the I^2^ statistic in *metan* |
| Description of statistical methods (eg complete description of fixed or random effects models, justification of whether the chosen models account for predictors of study results, dose-response models, or cumulative meta-analysis) in sufficient detail to be replicated | 11 | Paragraph stating that “where quantitative synthesis was possible, random effects meta-analysis was performed using the command metan in STATA (Version 14). Heterogeneity was assessed using the I^2^ statistic.” |
| Provision of appropriate tables and graphics | N/A |  |
| **Reporting of results should include** | | |
| Graphic summarizing individual study estimates and overall estimate | Figure 9 |  |
| Table giving descriptive information for each study included | Table 5 |  |
| Results of sensitivity testing (eg subgroup analysis) | N/A | No subgroup analysis performed. |
| Indication of statistical uncertainty of findings | Figure 9 |  |
| **Reporting of discussion should include** | | |
| Quantitative assessment of bias (eg publication bias) | Page 27 |  |
| Justification for exclusion (eg exclusion of non-English language citations) | N/A | No studies were excluded based upon non-English language |
| Assessment of quality of included studies | N/A | As this was a scoping review, no studies were excluded based upon quality. |
| **Reporting of conclusions should include** | | |
| Consideration of alternative explanations for observed results | Pages 24-26 |  |
| Generalization of the conclusions (eg appropriate for the data presented and within the domain of the literature review) | Pages 24-26 |  |
| Guidelines for future research | Page 27 |  |
| Disclosure of funding source | Page 28 |  |

Transcribed from the original paper within the Support Unit for Research Evidence (SURE), Cardiff University, United Kingdom. February 2011.
