# Supplementary figures and images for: The effect of prone positioning on maternal haemodynamics and fetal wellbeing in the third trimester–A primary cohort study with a scoping review
Source: PLoS One. 2023 Oct 11;18(10):e0287804. doi: 10.1371/journal.pone.0287804 (PMC10566740; doi:10.1371/journal.pone.0287804)

**Supplementary Figure 1**: Anna cushion by Karli Buchling.


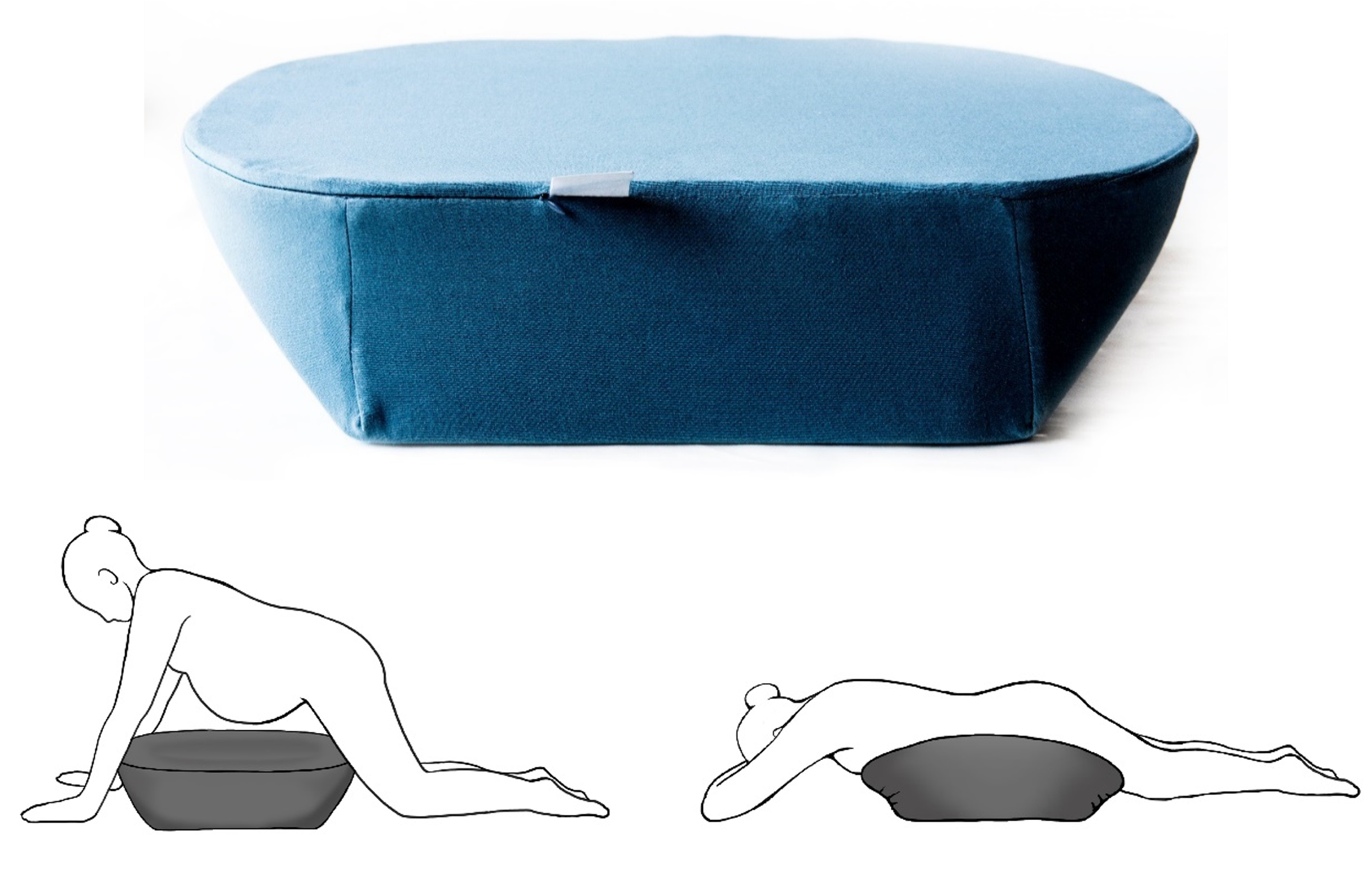

Supplement: S1 Fig — (DOCX) [file pone.0287804.s005.docx]
