# Supplementary material for: The effect of prone positioning on maternal haemodynamics and fetal wellbeing in the third trimester–A primary cohort study with a scoping review
Source: PLoS One. 2023 Oct 11;18(10):e0287804. doi: 10.1371/journal.pone.0287804 (PMC10566740; doi:10.1371/journal.pone.0287804)
Supplement: S1 File — (DOCX) [file pone.0287804.s006.docx]

**Supplementary Information – Example search strategy for the systematic review of prone position**

s1 exp pregnancy/

s2 maternal health/

s3 (pregnan$ or “maternal health”).mp.

s4 (health adj2 mother$).mp.

s5 ((maternal or mother$) adj2 (cardiorespirat$ or cardio-respirat$ or respirat$ or breathing or cardiac or heart or cardio$ or covid or coronavirus or surg$)).mp.

s6 exp fetal monitoring

s7 fetus/

s8 maternal-fetal exchange/

s9 placental circulation/

s10 (fetal OR fetus OR placenta$).mp.

s11 maternal-fetal exchange.mp.

s12 s1 OR s2 OR s3 OR s4 OR s5 OR s6 OR s7 OR s8 OR s9 or s10 OR s11

s13 prone position/

s14 prone position$.mp.

s15 patient position$ adj2 prone.mp.

s16 lying face down.mp.

s17 s13 OR s14 or s15 or s16

s18 s12 AND s18
