# Supplementary material for: The effect of prone positioning on maternal haemodynamics and fetal wellbeing in the third trimester–A primary cohort study with a scoping review
Source: PLoS One. 2023 Oct 11;18(10):e0287804. doi: 10.1371/journal.pone.0287804 (PMC10566740; doi:10.1371/journal.pone.0287804)
Supplement: S2 File — (DOCX) [file pone.0287804.s007.docx]

Records identified through database searching
(n = 639)

## Screening

## Included

## Eligibility

## Identification

Additional records identified through other sources
(n = 4)

Records after duplicates removed
(n = 478)

Records screened
(n = 478)

Records excluded
(n = 307)

Full-text articles assessed for eligibility
(n = 132)

Full-text articles excluded, with reasons (n =53)

- No explicit information or outcome data regarding prone position in pregnant participants (26)
- Prone position in post-partum period (15)
- Prone position considered by authors but not utilised (4)
- Prone position not discussed (1)
- Duplicate conference abstract or study protocol for study already included (4)
- Could not access (2)
- Review containing case report data already included (1)

Studies included in qualitative synthesis
(n =79)

Primary haemodynamic studies (n=3)

Observational or population study (n=14)

Case Studies (n= 44)

Review article or guideline (n=16)

Comment or opinion (n=5)
