# Supplementary material for: The effect of prone positioning on maternal haemodynamics and fetal wellbeing in the third trimester–A primary cohort study with a scoping review
Source: PLoS One. 2023 Oct 11;18(10):e0287804. doi: 10.1371/journal.pone.0287804 (PMC10566740; doi:10.1371/journal.pone.0287804)
Supplement: S3 File — (PDF) [file pone.0287804.s008.pdf]

Manuscript	No. Cases	Pathology	Gestation	Duration Prone (h)	Method of Proning	FHR monitor during	Adverse Outcome	Livebirth	Preterm	Maternal oxygenation	Fetal distress during prone
1	3	CES	24, 27, 30	3.5	Jackson table	Y	N	Y	N, Y, Y		N
2	3	CES	20, 20 16		Relton-Hall frame	N	N	Y	N, N, N		N
3	1	epidural haematoma	20		Wilson frame	after	N	Y	N		
4	2	lumbar fracture	17, 12		spinal bolsters	after	N	Y	N		
5	1	brain lesion	21	6.5	spinal bolsters	before and after	N	Y	N		
6	1	spinal lesion	32	2.5	three-quarters prone	Y	N	Y	N		N
7	1	CES	15	1	Jackson table	N	Y	Y, N	N		
8	1	CES	28		knee-chest position		N	Y			
9	1	Spinal fracture	26		Toronto frame	Y	N	Y	N		N
10	1	brain lesion	34		Three-quarters prone	Y	N	Y	Y		N
11	1	lumbar disc herniation	17		spinal bolsters	before and after	N	Y	N		
12	1	CES	25				N	Y	N		
13	1	spinal lesion	28	5	spinal bolsters	N	N				
14	1	CES	20-22		three-quarters prone	Y	N	Y	Y		N
15	1	CES	30			Y	N	Y	N		N
16	1	cord prolapse	37	0.1	knee-chest position	Y	N	Y	N	increase	N
17	2	lumbar disc herniation	11, 11			N	N	Y/N	N, N/A		
18	1	spinal lesion	12			N	N	Y	N		
19	1	CES	21		spinal bolsters	after	N	Y	N	no change	
20	1	CES	32				N	Y	N		
21	1	spinal fracture	17	4	Jackson table	N	N	Y	N		
22	1	CES	27+4	3	Jackson table	before and after	N	Y	N	no change	
23	1	spinal lesion	17	5		before and after	Y				
24	1	H1N1 Influenza ARDS	27			Y	N	Y	N	no change	N

25	1	COVID-19 ARDS	26	16-18 daily		Y	N	Y	Y	increase	N
26	1	Influenza A ARDS	26+6				N	Y	Y	no change	
27	1	COVID-19 ARDS	24	16 daily		Y	N			increase	N
28	1	COVID-19 ARDS	27	16	padded supports + mattress deflated at abddomen	before and after	N			increase	
29	1	chest trauma	34	8	padded supports	Y	N	Y	N	increase	N
30	1	COVID-19 ARDS	25			before and after	N			increase	N
31	1	COVID19 ARDS	27+4	12 daily	padded supports	Y	N	Y	Y	increase	N
32	1	ARDS	10								
33	1	Influenza A ARDS	23		padded supports	Y	N	Y	Y	increase	N
34	1	H1N1 Influenza ARDS	21	16+ daily	padded supports	Y	N			increase	N
35	1	COVID-19 ARDS	23+6		RotoProne bed and doughnut		N	Y	Y	increase	N
36	7	renal stones	12	1.4 (mean)			N	Y	N		
37	1	sacroiliac joint pain	21				N				
38	1	scrub typhus ARDS	20	48	padded supports	Y	N			increase	N
39	1	COVID-19 ARDS	22+6	2 cycles of 24 + 1 48 cycle			N	Y	Y	increase	N
40	4	ARDS	24+6,25,27+6,29	16 +14, 16, 16,1-16.15	self-proning, A-frame supports	Y	N	Y	N,N,Y,N	increase	N
41	1	COVID-19 ARDS	28+6	4+ daily	Pillows and foam supports	Y	N	Y	N	increase	N
42	2	spinal surgery	10,19	60+			N	Y	N,Y		
43	1	COVID-19 ARDS	24+3	16+ daily		Y	N	Y	Y	increase	N
44	13	COVID-19 ARDS		16 (mean)		Y	N			no change	N

45	1	COVID-19 ARDS	27+5	8+ daily	padded supports + pillows + blankets		N			increase	
46	1	disc herniation	17					Y	N		
47	1	CES	30	2.5	Jackson table	Y	N	Y	N		N
