# Supplementary material for: The effect of prone positioning on maternal haemodynamics and fetal wellbeing in the third trimester–A primary cohort study with a scoping review
Source: PLoS One. 2023 Oct 11;18(10):e0287804. doi: 10.1371/journal.pone.0287804 (PMC10566740; doi:10.1371/journal.pone.0287804)
Supplement: S4 File — (PDF) [file pone.0287804.s009.pdf]

RESEARCH PROTOCOL

Does the Use of a Cushion to Support Maternal Prone Position during Therapy affect Maternal Cardiorespiratory Status or the Fetal Heart Rate?

Contents

1)	RESEARCH TEAM & KEY CONTACTS	3
2)	INTRODUCTION	4
3)	BACKGROUND	5
4)	STUDY OBJECTIVES	7
5)	STUDY DESIGN & PROTOCOL	7
6)	STUDY PARTICIPANTS	8
7)	OUTCOME MEASURES	9
8)	DATA COLLECTION, SOURCE DATA AND CONFIDENTIALITY	10
9)	STATISTICAL CONSIDERATIONS	10
10)	DATA MONITORING AND QUALITY ASSURANCE	11
11)	ETHICAL CONSIDERATIONS	7
12)	STATEMENT OF INDEMNITY	12
13)	FUNDING	12
14)	PUBLICATION POLICY	12
15)	REFERENCES	12

1) RESEARCH TEAM & KEY CONTACTS

Chief Investigator:	***Co-investigator(s):***
Name: Professor Alexander Heazell<br><br>Address:<br>Maternal and Fetal Health Research Centre<br>5th Floor Research, St. Mary's Hospital<br>Oxford Road, Manchester<br>M13 9WL<br><br>Email: alexander.heazell@manchester.ac.uk<br><br>Telephone: (0161) 276 6484<br><br>Fax:	Name: Karli Büchling<br><br>Address:<br>54 Beceshore Close,<br>Moreton-in-Marsh,<br>GL56 9NB<br><br>Email: karli.buchling@gmail.com<br><br>Telephone: 07713 454962<br><br>Fax:
Sponsor(s):<br><br>Name: The University of Manchester<br><br>Sponsor Contact: Ms Lynne Macrae, Faculty Research Practice Governance Coordinator<br><br>Address:<br>Faculty of Biology, Medicine and Health<br>5.012 Carys Bannister Building<br>University of Manchester<br>M13 9PL<br><br>Email: FBMHethics@manchester.ac.uk<br><br>Telephone: 0161 275 5436	***Lead R&D Trust contact(s):***<br><br>Name: Manchester University NHS Foundation Trust<br><br>Contact: Dr Elizabeth Mainwaring<br><br>Address:<br>Manchester University NHS Foundation Trust<br>Research Office,<br>Manchester University NHS Foundation Trust<br>29 Grafton Street, Manchester, M13 9WU<br><br>Email: R&D.applications@mft.nhs.uk<br><br>Telephone: 0161 276 3340

2) INTRODUCTION (IRAS A6-1)

Lay Summary

During pregnancy women may need or choose to undergo physical therapies such as physiotherapy, massage or osteopathy. Recent findings from studies of mothers who had a stillbirth in late pregnancy found that the position in which women went to sleep in was linked to stillbirth, as was the frequency of day time naps. This link is thought to be due to changes in mother's blood flow from her heart when lying flat leading to changes in the amount of oxygen going to her baby. This raise concerns that spending extended periods laid flat could be detrimental to baby's health. However, it is not known whether lying flat for extended periods for physical therapies could also alter a baby's heart rate or levels of oxygen. One small study of 33 women from Brazil found that there were no differences in a mother's heart rate, blood pressure, oxygen saturation or baby's heart rate. But there were changes in mother's breathing rate and systolic blood pressure when a mother laid on her front. All the women reported feeling comfortable lying flat (on a bent surface). However, in this study women only spent 6 minutes in each position which is less than a woman would be expected to spend lying in a position for a session of physical therapy. We plan a study to assess whether using a device to support a prone position (Anna cushion) would be associated with changes in mother's heart rate, blood pressure, breathing rate and blood oxygen levels and baby's heart rate. We will also ask about mother's levels of comfort while she is laid in the prone position. The findings of this study will give an indication whether supporting a mother to lie in a prone position for physical therapies is safe and comfortable.

Scientific Summary

Women frequently experience lower-back or pelvic pain during pregnancy. This may lead to a need for physical therapies such as physiotherapy, osteopathy or massage in late pregnancy. Several case-control studies, and a recent individual patient data meta-analysis has demonstrated an association between going to sleep position and late stillbirth (a greater than 2-fold increased risk with going to sleep supine) and increased frequency of daytime naps. This is thought to be related to maternal haemodynamic changes when a mother lies supine in late pregnancy which decreases cardiac output and uterine blood flow. These changes are accompanied by alterations in fetal behaviour which are consistent with a reduction in oxygenation. This observation raises concerns that spending extended periods laid flat could be detrimental to baby's health. However, it is not known whether lying flat for extended periods for physical therapies could also alter a baby's heart rate or levels of oxygen. One small study of 33 women from Brazil which randomised the order of maternal positions found that there were no differences in a mother's heart rate, blood pressure, oxygen saturation or baby's heart rate between a supine, lateral and prone position (bent over a concave couch). However, there were observed changes in mother's breathing rate and systolic blood pressure when a mother laid on her front. Nevertheless, all the women reported feeling comfortable lying flat (on a bent surface). However, in this study women only spent 6 minutes in each position which is less than a woman would be

expected to spend lying in a position for a session of physical therapy. Therefore, further work is required to determine whether spending extended periods laid prone is safe for mother and baby.

The co-investigator (Karli Büchling) has developed a device to support mothers in a prone position (Anna cushion). This study will investigate whether adopting this position supported by Anna cushion is associated with changes in mother's heart rate, blood pressure, breathing rate and blood oxygen levels and fetal heart rate as assessed by the cardiotocograph. We will also ask about mother's levels of comfort while she is laid flat. The findings of this study will give an indication whether supporting a mother to lie in a prone position for physical therapies is safe and comfortable

3) BACKGROUND (IRAS A12)

The association between maternal position and cardiac output has been known for many years [1]. More recently, studies have demonstrated an association between the position in which a mother goes to sleep and late stillbirth (after 28 weeks) [2-6]. This has been consistently demonstrated in five studies from four different countries. Furthermore, there is an association between the frequency of daytime naps and the risk of late stillbirth [2, 3]. This is hypothesised to be due to frequent exposure to a supine sleeping position. When a mother lies flat there is a reduction in cardiac output and consequent uterine blood flow, this is due to compression of the inferior vena cava by the gravid uterus [1, 7]. These changes are associated with alterations in fetal behaviour consistent with a reduction of fetal oxygenation [8]. Whilst sleep is associated with extended periods spent in specific positions little is known about the effect of maternal position for other purposes in late pregnancy.

One study of maternal position exposed 33 women to a supine, lateral and prone positions in a random sequence [9]. To maintain a prone position woman used a concave stretcher designed. This study adopted each position for 6 minutes following a 10-minute period of adjustment to the experimental surroundings. This study found no differences in a mother's heart rate, diastolic blood pressure, oxygen saturation or baby's baseline heart rate between supine, lateral and prone positions (bent over a concave couch) [9]. However, there were changes in mother's respiratory rate and systolic blood pressure when a mother laid prone. Nevertheless, all the women reported feeling comfortable lying flat (on a bent surface). A more recent study of 50 healthy pregnant women and 15 women with preeclampsia found that lying in a prone position for 5 minutes prior to observations was associated with a reduction in systolic blood pressure, which for women with preeclampsia could be beneficial [10]. Importantly, there have not been any studies investigating a more clinically meaningful timeframe of exposure of adopting a supine position for physical therapies e.g. 30 minutes. Therefore, further studies are required.

Clearly, maintaining a prone position for physical therapies in late pregnancy is difficult due to the gravid uterus. Co-investigator (Karli Büchling) has developed a device for to support mothers in a prone position

(Anna cushion). Anna cushion (Figure 1) is designed to allow pregnant women to lie safely and comfortably in a prone position up until the end of their pregnancy. Anna cushion itself is specifically moulded from of a medical grade, medium density, closed cell foam, which has been covered in a double layer of cotton interlock fabric. The height of the cushion is 15cm, with the inner section having been cut out and contoured to support the pregnant abdomen. The fabric covering has enough stretch to support the abdomen, whilst not restricting movement in any way. The length of the cushion is 68 cm, with tapered caudal and distal ends, to allow the woman to lie comfortably, without any unnecessary pressure exerted on the chest or hips.

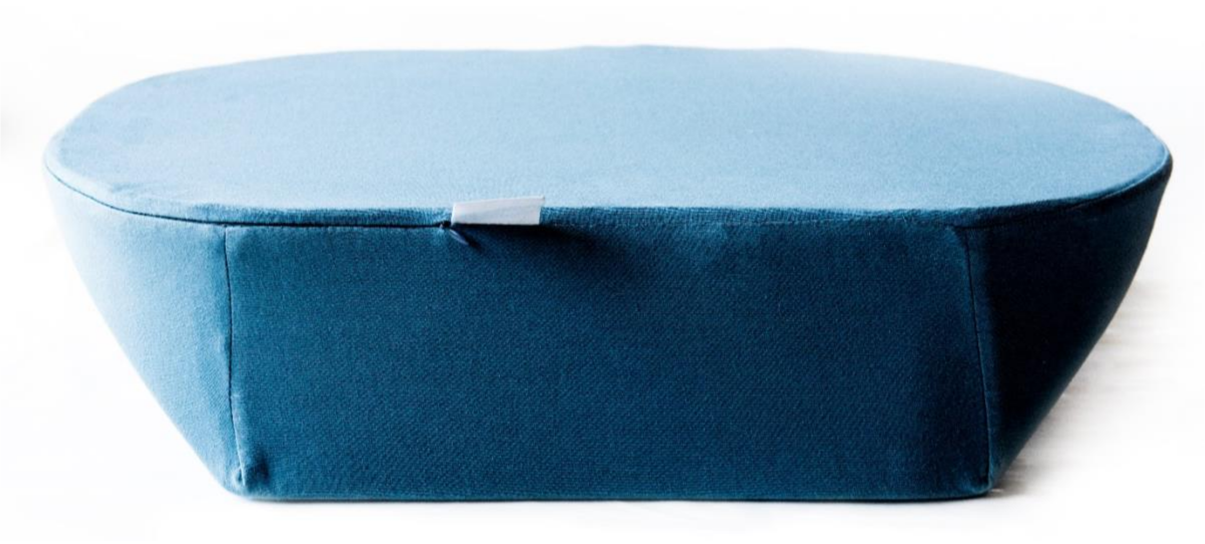

Figure 1 Anna cushion

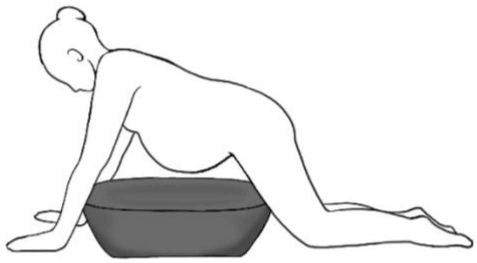	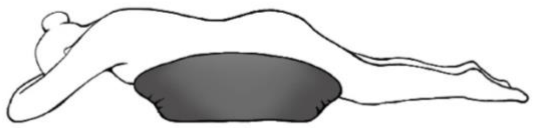
The patient positions themselves over Anna cushion, placing their hands/arms in front of the cushion, their bump over the middle section of the cushion and their knees behind the cushion.	The patient lowers themselves down carefully so that their bump falls neatly in the middle of the carved-out section, their breasts touching the top end of the cushion, and the top of their legs resting on the bottom end of the cushion.

Table 1 How to use Anna cushion

The proposed study will investigate whether adopting this position supported by Anna cushion for a clinically meaningful timeframe is associated with changes in mother's heart rate, blood pressure, cardiac output, breathing rate and blood oxygen levels and fetal heart rate as assessed by continuous cardiotocography. We will also ask about mother's levels of comfort while she is laid flat. The findings of this study will give an indication whether supporting a mother to lie in a prone position for physical therapies is safe and comfortable.

4) STUDY OBJECTIVES

4.1 Primary Question/Objective: (IRAS A10)

To describe the cardiorespiratory effects for a mother maintaining a prone position supported by the cushion (Anna cushion) for a period of 30 minutes.

4.2 Secondary Question/Objective: (IRAS A11)

To determine whether maintaining a prone position is associated with any effects on the fetal heart rate, and to determine whether using a device to support a prone position is comfortable for the mother.

5) STUDY DESIGN & PROTOCOL

5.1 Participants (IRAS A15)

We will conduct a cohort study of women with a viable singleton pregnancy over 28 weeks' gestation.

5.2 Study Intervention and/or Procedures (IRAS A13, A18, A19)

Prior to commencing the study women will be asked to complete a short questionnaire about their pregnancy which will ask about symptoms of pain or discomfort, their anxiety levels and prior knowledge and experience of maternal position during sleep or rest. They will then have fetal and maternal monitoring connected via a series of self-adhesive electrodes. The fetal heart rate and uterine activity will be monitored using the MONICA AN24 system which has been used previously by our group to monitor fetal heart rates for extended periods [11]. This involves application of five self-adhesive ECG electrodes to the maternal abdomen (Figure 2).

[redacted]

Figure 2 – MONICA AN24 fetal monitoring system showing electrodes applied to

Maternal cardiac output, heart rate and blood pressure will be measured by the Cheetah NICOM system which has self-adhesive electrodes applied to the maternal back and a blood pressure cuff to the maternal upper arm. The NICOM system has previously been validated for use in pregnancy [12]. A pulse oximetry probe will be applied to the left forefinger. The mother will be asked to lie

on her left-hand side (the optimal position for maternal and fetal wellbeing) for 20 minutes with observations recorded at 5-minute intervals on to a case report form.

If any of the maternal or fetal values are outside of the anticipated range the experimental protocol will be discontinued. Women who have hypertension (>140/90 mmHg), tachycardia (>110 bpm), tachpnoea (>20 breaths per minute) or abnormal fetal heart rate traces will be referred to the maternity service for further assessment. A transfer letter template has been devised to rapidly report problems to the receiving medical team.

If the maternal and fetal observations are normal during the period spent in left lateral position the mother will be positioned in the prone position on the device for a period of 30 minutes. During this time observations recorded at 5-minute intervals on to a case report form. If at any point the mother feels unwell or the observations become abnormal (as outlined above) the experimental protocol will be ceased and the mother returned to a left lateral position. If there are persistent abnormalities of the fetal heart rate trace the mother will be referred urgently to the maternity service.

Following a 30-minute period in the prone position participants will then be returned to the left lateral position for 20 minutes of further monitoring with observations recorded at 5-minute intervals. After this period the monitoring will be disconnected and the electrodes removed. The site will be checked for signs of skin irritation. Women will then be asked to complete a short questionnaire to describe their experience of using the device and a final survey of their anxiety levels. Women will then be given a small token of thanks for their participation.

6) STUDY PARTICIPANTS

The local research midwives and nurses will be trained to discuss specific aspects of this study, to obtain informed consent and apply the electrodes and blood pressure cuff for the MONICA AN24 device and the NICOM system. The local Principal Investigator, Chief Investigator and co-investigator can be contacted if there are any concerns regarding adherence to the protocol and to deal with any issues arising from the investigations.

6.1 Inclusion Criteria: (IRAS A17-1)

- Viable singleton pregnancy ⩾28 weeks’ gestation
- No fetal anomalies according to Fetal Anomaly Screening Programme definition
- Ability to give written informed consent
- Maternal Age >16 years

6.2 Exclusion Criteria: (IRAS A17-2)

- Unable to read English (as the survey instruments are only available in English)
- Multiple pregnancy
- Pre-existing maternal cardiovascular or respiratory disease
- Fetal anomaly
- Contraindication to lying prone (severe pain etc.)
- Allergy to self-adhesive electrodes used for standard electrocardiography (ECG)
- Unable to give written informed consent

6.3 Recruitment: (IRAS A27-1 – A35)

We will conduct a cohort study of women with a viable singleton pregnancy over 28 weeks' gestation. Women at low-risk of complications will initially be approached in routine antenatal clinics from 20 weeks' gestation, women who agree to hear more about the study will then be given brief information about the study and will then be asked to complete a consent to contact form to allow the researchers to contact them at an appropriate point in their pregnancy. If women are willing to participate in the study when they are contacted, they will be sent a participant information sheet. Written consent will be taken prior to participating in the study.

6.5 Participants who withdraw consent [or lose capacity to consent]:

Participants can withdraw consent at any time without giving any reason, as participation in the research is voluntary, without their care or legal rights being affected. For participants who voluntarily withdraw from the study who have previously consented to the study data up to the time of withdrawal will be included in the study unless the participants specifically request for all of their data be removed.

As participation in the study is limited to a single window during which participants will have a period of monitoring capacity to consent will be continuously monitored. After this period of contact ongoing capacity will not be assessed and participants' data will be included in the study unless participants specifically request for their data to be removed.

7) OUTCOME MEASURES (IRAS A57, A58)

The study is anticipated to last for six months (at a recruitment rate of two participants per week) and will commence in January 2020. It is estimated that data cleaning and analysis will take an additional three months.

Primary Outcome Measure

Maternal cardiac output as assessed by non-invasive cardiac monitoring.

Secondary Outcome Measures

A variety of other measures of maternal cardio-respiratory status will be measured, including: heart rate, blood pressure, respiratory rate and oxygenation via pulse oximetry.

Fetal wellbeing assessed by continuous cardiotocography which will report baseline heart rate, variability, the presence of accelerations or decelerations.

Acceptability, including maternal anxiety, will be assessed using standardised measures and comfort using a user-completed questionnaire.

8) DATA COLLECTION, SOURCE DATA AND CONFIDENTIALITY (IRAS A36-A45)

The study will be carried out at single tertiary maternity unit (St Mary's Hospital, Manchester). Prof Alexander Heazell will be the Chief Investigator and the Principal Investigator at the St Mary's site. The day-to-day running of the project will be carried out by the local research midwife or nurse from the participating unit. Any problems or queries will be passed to the Principal Investigator in the first instance. In their absence the Chief investigator will deputise.

Data will be initially collected on written case report forms by the local research midwives/nurses who will then input the information onto a secure database (in RedCap). All records will be double entered to check for validity. The case report forms will be stored as the original study record in a dedicated file.

All study data will be entered into a database customised for the study in which the participant will be identified only by a specific study number. The participants name and other identifiable data will be stored in a separate database linked only by the study number. All data generated by the study will be preserved and stored with no patient identifiable details for 5 years after completion of the study. Prof Alexander Heazell will be custodian of the data on behalf of the Sponsor. Electronic data will be stored in appropriate data storage devices. All electronic and hard-copy data files will be stored securely in accordance with local policy. Storage of electronic data will be on password protected files on a restricted area of a file server which is in a secure location. Data will be processed on a workstation by authorised staff using passwords.

9) STATISTICAL CONSIDERATIONS

9.1 Statistical Analysis (IRAS A56, A62)

Data regarding maternal characteristics will be analysed using descriptive statistics. Data regarding maternal cardiac output in different maternal positions will initially be evaluated to determine whether values are normally distributed. Depending on the distribution of values, they will be transformed to a normal distribution if appropriate and a repeated measures ANOVA performed. If they are not normally distributed and transformation does not achieve a normal distribution a Friedman test will be performed. This approach will address variation between individual participants.

Descriptive and comparative statistical analysis will be conducted in STATA (STATACORP, TX, USA). As the investigators anticipate some variation in the baseline observations between participants both raw values and change from baseline status will be evaluated.

9.2 Sample Size: (IRAS A59, A60)

The sample size for this study has been calculated to determine whether maternal cardiac output (the primary outcome measure) decreases in the prone position compared to left lateral position. Milsom et al. demonstrated a mean cardiac output of 6.6L/min in left lateral position, 5.9L/min in right lateral position and 5.5L/min in a supine position and there was a standard deviation of 1.0L/min [1]. To have 80% power to detect a fall from the level reported for left lateral position (6.6 L/min) to 5.7 L/min (the midpoint between right lateral and supine positions) 20 participants would be required in each group using alpha=0.05.

10) DATA MONITORING AND QUALITY ASSURANCE (IRAS A74, A75-1)

The study will be subject to the audit and monitoring regime of the University of Manchester. The investigators / institutions will facilitate study-related monitoring, audits, review by independent ethics committee and regulatory inspections providing direct access to source data / documents to authorised study or regulatory personnel.

11) SAFETY CONSIDERATIONS AND ADVERSE EVENTS (IRAS A75-1, A75-2)

Prior studies suggest that lying prone does not have adverse consequences for mother or baby.[9] However, it is possible that a mother may develop symptoms, have deviations from blood pressure or that the fetal heart rate trace would become abnormal. If these situations develop during the experimental protocol the procedure would be stopped and the mother transferred to a left-lateral position to allow a period for the abnormal symptoms or observations to resolve. The mother would be clinically assessed by the research midwife, if symptoms or abnormal observations do not resolve the mother would be transferred to the maternity department at St Mary's Hospital for further management. A handover would be given to the clinical staff by the research midwife looking after the participant. The need to terminate the protocol would be recorded in the study and reported in the final analysis.

12) PEER REVIEW (IRAS A54-1)

This protocol has been peer-reviewed within the investigators institution by members with existing expertise on measurement of cardiac output and haemodynamic indices during pregnancy. Statistical input has been sought from Prof Jenny Myers, Maternal and Fetal Health Research Centre.

13) ETHICAL and REGULATORY CONSIDERATIONS

13.1 Approvals

Health Research Authority approval will be obtained before commencing research processes. The Medicines and Health Regulatory Agency have confirmed that the Anna Pillow does not need a CE mark to be used in practice or research as it is primarily concerned with comfort or pain relief.

The study will be conducted in full conformance with all relevant legal requirements and the principles of the Declaration of Helsinki, Good Clinical Practice (GCP) and the UK Policy Framework for Health and Social Care Research 2017.

13.2 Risks (IRAS A22 and A26)

There is no risk to researchers, local health and safety SOPs will be followed at the Manchester Maternal and Fetal Health Research Centre.

14) STATEMENT OF INDEMNITY (IRAS A76-1,-2,-3, A77)

The University has insurance available in respect of research involving human subjects that provides cover for legal liabilities arising from its actions or those of its staff or supervised students. The University also has insurance available that provides compensation for non-negligent harm to research subjects occasioned in circumstances that are under the control of the University.

15) FUNDING and RESOURCES (IRAS A65)

The investigators have prepared applications to several charitable organisations (including the Dowager Countess Eleanor Peel Trust and the Sir Halley Stewart Research Trust). The results of these applications are awaited.

16) PUBLICATION POLICY (IRAS A50-1 – A53)

After Research Ethics Committee and HRA approval has been obtained this protocol will be registered on www.clinicaltrials.gov. The investigators will disseminate the results of this study at appropriate national and international scientific meetings such as the British Maternal Fetal Medicine Society. Primary responsibility for preparing publications will lie with the Chief Investigator, Prof Alexander Heazell. To safeguard the integrity of the study, data from the study will not be presented in public before the main results are published without the prior consent of the co-investigators. Acknowledgements will include the trial staff at the study site.

17) REFERENCES

1. Milsom, I. and L. Forssman, *Factors influencing aortocaval compression in late pregnancy.* Am J Obstet Gynecol, 1984. **148**(6): p. 764-71.

2. Heazell, A., M. Li, J. Budd, J. Thompson, T. Stacey, R.S. Cronin, B. Martin, D. Roberts, E.A. Mitchell, and L. McCowan, *Association between maternal sleep practices and late stillbirth - findings from a stillbirth case-control study.* BJOG, 2018. **125**(2): p. 254-262.

3. Stacey, T., J.M. Thompson, E.A. Mitchell, A.J. Ekeroma, J.M. Zuccollo, and L.M. McCowan, *Association between maternal sleep practices and risk of late stillbirth: a case-control study.* BMJ, 2011. **342**: p. d3403.

4. Gordon, A., C. Raynes-Greenow, D. Bond, J. Morris, W. Rawlinson, and H. Jeffery, *Sleep position, fetal growth restriction, and late-pregnancy stillbirth: the Sydney stillbirth study.* Obstet Gynecol, 2015. **125**(2): p. 347-55.

5. Owusu, J.T., F.J. Anderson, J. Coleman, S. Oppong, J.D. Seffah, A. Aikins, and L.M. O'Brien, *Association of maternal sleep practices with pre-eclampsia, low birth weight, and stillbirth among Ghanaian women.* Int J Gynaecol Obstet, 2013. **121**(3): p. 261-5.

6. McCowan, L.M.E., J.M.D. Thompson, R.S. Cronin, M. Li, T. Stacey, P.R. Stone, B.A. Lawton, A.J. Ekeroma, and E.A. Mitchell, *Going to sleep in the supine position is a modifiable risk factor for late pregnancy stillbirth; Findings from the New Zealand multicentre stillbirth case-control study.* PLoS One, 2017. **12**(6): p. e0179396.

7. Humphries, A., S.A. Mirjalili, G.P. Tarr, J.M.D. Thompson, and P. Stone, *The effect of supine positioning on maternal hemodynamics during late pregnancy.* J Matern Fetal Neonatal Med, 2018: p. 1-8.

8. Stone, P.R., W. Burgess, J. McIntyre, A.J. Gunn, C.A. Lear, L. Bennet, E.A. Mitchell, and J.M.D. Thompson, *An investigation of fetal behavioural states during maternal sleep in healthy late gestation pregnancy: an observational study.* J Physiol, 2017. **595**(24): p. 7441-7450.

9. Oliveira, C., M.A.B. Lopes, A.S. Rodrigues, M. Zugaib, and R.P.V. Francisco, *Influence of the prone position on a stretcher for pregnant women on maternal and fetal hemodynamic parameters and comfort in pregnancy.* Clinics (Sao Paulo), 2017. **72**(6): p. 325-332.

10. Dennis AT, Hardy L, Leeton L. *The prone position in healthy pregnant women and in women with preeclampsia - a pilot study.* BMC Pregnancy Childbirth 2018 Nov 16;18(1):445. doi: 10.1186/s12884-018-2073-x.

11. Crawford, A., P. Anyadi, L. Stephens, S.L. Thomas, H. Reid, L.E. Higgins, L.K. Warrander, E.D. Johnstone, and A.E.P. Heazell, *A mixed-methods evaluation of continuous electronic fetal monitoring for an extended period.* Acta Obstet Gynecol Scand, 2018. **97**(12): p. 1515-1523.

12. Vinayagam, D., O. Patey, B. Thilaganathan, and A. Khalil, *Cardiac output assessment in pregnancy: comparison of two automated monitors with echocardiography.* Ultrasound Obstet Gynecol, 2017. **49**(1): p. 32-38.
