# Supplementary material for: The effect of prone positioning on maternal haemodynamics and fetal wellbeing in the third trimester–A primary cohort study with a scoping review
Source: PLoS One. 2023 Oct 11;18(10):e0287804. doi: 10.1371/journal.pone.0287804 (PMC10566740; doi:10.1371/journal.pone.0287804)
Supplement: S6 File — (PDF) [file pone.0287804.s011.pdf]

Ymchwil Iechyd a Gofal Cymru
Health and Care Research Wales

NHS
Health Research Authority

Prof Alexander Heazell
Professor of Obstetrics
Division of Developmental Biology and Medicine,
School of Medical Sciences, University of Manchester
5th Floor St. Mary's Hospital, Oxford Road
Maternal and Fetal Health Research Centre
Manchester
M13 9WL

Email: approvals@hra.nhs.uk
HCRW.approvals@wales.nhs.uk

19 January 2021

Dear Prof Heazell

HRA and Health and Care Research Wales (HCRW) Approval Letter

**Study title:**	**Does Maternal Prone Position affect Maternal Cardiorespiratory Status or the Fetal Heart Rate?**
**IRAS project ID:**	**240071**
**REC reference:**	**20/NE/0261**
**Sponsor**	**University of Manchester**

I am pleased to confirm that **HRA and Health and Care Research Wales (HCRW) Approval** has been given for the above referenced study, on the basis described in the application form, protocol, supporting documentation and any clarifications received. You should not expect to receive anything further relating to this application.

Please now work with participating NHS organisations to confirm capacity and capability, in line with the instructions provided in the "Information to support study set up" section towards the end of this letter.

How should I work with participating NHS/HSC organisations in Northern Ireland and Scotland?
HRA and HCRW Approval does not apply to NHS/HSC organisations within Northern Ireland and Scotland.

If you indicated in your IRAS form that you do have participating organisations in either of these devolved administrations, the final document set and the study wide governance report (including this letter) have been sent to the coordinating centre of each participating nation. The relevant national coordinating function/s will contact you as appropriate.

Please see IRAS Help for information on working with NHS/HSC organisations in Northern Ireland and Scotland.

How should I work with participating non-NHS organisations?
HRA and HCRW Approval does not apply to non-NHS organisations. You should work with your non-NHS organisations to obtain local agreement in accordance with their procedures.

What are my notification responsibilities during the study?

The standard conditions document "*After Ethical Review – guidance for sponsors and investigators*", issued with your REC favourable opinion, gives detailed guidance on reporting expectations for studies, including:

- Registration of research
- Notifying amendments
- Notifying the end of the study

The HRA website also provides guidance on these topics, and is updated in the light of changes in reporting expectations or procedures.

Who should I contact for further information?
Please do not hesitate to contact me for assistance with this application. My contact details are below.

Your IRAS project ID is **240071**. Please quote this on all correspondence.

Yours sincerely,
Steph Blacklock

Approvals Manager

Email: approvals@hra.nhs.uk

Copy to: *Ms Lynne MacRae, Sponsor Contact*

List of Documents

The final document set assessed and approved by HRA and HCRW Approval is listed below.

Document	*Version*	*Date*
Contract/Study Agreement template [University of Manchester mNCA]		
Copies of advertisement materials for research participants [Participant advert]	1.0	24 July 2020
Evidence of Sponsor insurance or indemnity (non-NHS Sponsors only)		
GP/consultant information sheets or letters [GP letter]	1.0	22 September 2020
IRAS Application Form [IRAS_Form_23102020]		23 October 2020
Non-validated questionnaire [Questionnaire for prior to monitoring protocol]	1.0	24 September 2020
Non-validated questionnaire [Questionnaire for after the monitoring protocol]	1.0	24 September 2020
Organisation Information Document [OID]	1.0	22 October 2020
Other [Case Report form]	1.0	20 October 2020
Other [Evidence of Indemnity]		23 October 2020
Other [Consent to contact document]	1.0	22 September 2020
Other [Case Report form]	1.1	27 November 2020
Other [Model NCA]	1.0	08 March 2019
Other [Responses for REC/HRA questions]	1.0	05 January 2021
Participant consent form [Consent Form]	1.0	22 September 2020
Participant consent form [Consent Form]	1.1	27 November 2020
Participant information sheet (PIS)	1.0	20 October 2020
Participant information sheet (PIS) [Participant Information Sheet]	1.1	27 November 2020
Referee's report or other scientific critique report [Review of protocol - Professor Jane Warland]		06 November 2020
Research protocol or project proposal [Study Protocol]	1.0	20 October 2020
Research protocol or project proposal [Study Protocol]	1.1	27 November 2020
Schedule of Events or SoECAT [Validated Schedule of Events]	1	02 November 2020
Schedule of Events or SoECAT		
Summary CV for Chief Investigator (CI) [CI's CV]		23 October 2020
Summary of any applicable exclusions to sponsor insurance (non-NHS sponsors only)		

IRAS project ID	240071

Information to support study set up

The below provides all parties with information to support the arranging and confirming of capacity and capability with participating NHS organisations in England and Wales. This is intended to be an accurate reflection of the study at the time of issue of this letter.

Types of participating NHS organisation	Expectations related to confirmation of capacity and capability	Agreement to be used	Funding arrangements	Oversight expectations	HR Good Practice Resource Pack expectations
One site type. The single site will conduct all activities as per the protocol.	Research activities should not commence at participating NHS organisations in England or Wales prior to their formal confirmation of capacity and capability to deliver the study.	An organisation information document has been submitted and the sponsor is intending to use a separate site agreement. The agreement is unmodified.	Funding to sites will be as per the mNCA provided.	A Principal Investigator should be appointed at study sites of this type	No Honorary Research Contracts, Letters of Access or pre-engagement checks are expected for local staff employed by the participating NHS organisations. Where arrangements are not already in place, research staff not employed by the NHS host organisation undertaking any of the research activities listed in the research application would be expected to obtaina Letter of Access based on standard DBS checks and occupational health clearance.

Other information to aid study set-up and delivery

This details any other information that may be helpful to sponsors and participating NHS organisations in England and Wales in study set-up.
The applicant has indicated that they intend to apply for inclusion on the NIHR CRN Portfolio<br><br>The Anna Cushions will be covered by the indemnity for the study as these are being used to support the mother in a prone position. Indemnity is being sought from CJ Insurance with Â£1M of cover for fetal injury.
